# The Influence of Family Dog Ownership and Parental Perceived Built Environment Measures on Children’s Physical Activity within the Washington, DC Area

**DOI:** 10.3390/ijerph14111398

**Published:** 2017-11-16

**Authors:** Jennifer D. Roberts, Lindsey Rodkey, Cortney Grisham, Rashawn Ray

**Affiliations:** 1 Department of Kinesiology, School of Public Health, University of Maryland, College Park, MD 20742, USA; lmrodkey@umd.edu; 2 College of Science and Mathematics, Austin Peay State University, Clarksville, TN 37044, USA; cgrisham1@my.apsu.edu; 3 Department of Sociology, University of Maryland, College Park, MD 20742, USA; rjray@umd.edu

**Keywords:** dog ownership, physical activity, parental perception, built environment

## Abstract

Sedentary behavior and physical inactivity are significant contributors to youth obesity in the United States. Neighborhood dog walking is an outlet for physical activity (PA). Therefore, understanding the relationship between built environment, dog ownership, and youth PA is essential. This study examined the influence of dog ownership and parental built environment perceptions on children’s PA in the Washington, D.C. area. In 2014, questionnaires were mailed to 2000 parents to assess family dog ownership; children’s outdoor dog walking or playing; and parental perceived built environment measures. Chi-square analyses examined differences in parental perceived built environment measures between children with and without family dogs. The sample included 144 children (50% female; average-age 9.7 years; 56.3% White; 23.7% African-American; 10.4% Asian-American; 29.9% owned dog). Only 13% and 5.6% of the children walked or played outdoors with the dog daily, respectively. A significantly greater proportion (*p*-value < 0.05) of parents who owned dogs recognized and observed some home built environment measures (e.g., traffic speed on most streets is 30 mph or less) that were PA -promoting for their children. Findings suggest that dog ownership may provide more positive parental perceptions of the neighborhood built environment, which supports children’s outdoor PA through dog walking and playing.

## 1. Introduction

Sedentary behavior and physical inactivity are significant contributors to obesity in the United States. The Centers for Disease Control and Prevention reports that 36.5% of U.S. adults and 17% of youth have obesity [1]. Engaging in daily physical activity (PA), such as recreational sports and leisure activities including biking, jogging, and dog walking, can help combat obesity. Dog walking is a unique outlet for PA, considering that one-third of U.S. households are dog owners [2]. One of the first research studies that reported the health benefits of pet ownership was published out of the University of Cambridge over 25 years ago [3]. Since that time, nearly two dozen empirical studies examining the relationship between dog walking and PA have been published in the U.S. However, this entire body of research has focused on adults, with the exception of two studies that explored this relationship among adolescents 12 years of age and older, and one study among children 4–10 years of age [4,5,6]. Among these two U.S.-based studies in adolescents, it was found that dog ownership was associated with more PA among adolescents by way of walking or playing with the dog [4,5]. Likewise, in the one and only U.S.-based study in younger children, a higher level of child–dog attachment was found to be associated with a higher level of PA [6].

Research on adolescent PA and dog ownership performed outside the U.S. has reported mixed results. Specifically, in metropolitan Perth and nonmetropolitan regions in Western Australia, observational conclusions revealed that adolescents who walked or played with their dog spent an average of one hour per week on each activity and were significantly more likely to meet national PA recommendations when compared to adolescents who did not engage in these activities [7]. Yet, in the United Kingdom, no evidence was found to support a relationship between objectively measured adolescent PA, specifically dog walking, and dog ownership [8].

Other research on preadolescent PA and dog ownership performed outside the U.S. has reported more positive than negative results. When a preadolescent population of youth was observed in Melbourne, Australia, researchers detected an association between dog ownership and an increase in PA amounting to an additional 29 min per week among girls 5–6 years old and 59 more minutes in girls 10–12 years old [9]. Another cross-sectional Australian study observed similar positive associations between dog ownership and PA among children 10–12 years old [10]. Dog-owning children 9–10 years old from the Liverpool SportsLinx Project engaged in dog walking several times a week more compared to children without a dog [11]. Another study conducted in the United Kingdom found that children 9–10 years of age with a dog spent more time engaging in light and vigorous PA and recorded higher levels of PA counts per minute with by ActiGraph GT1M activity monitors than those without dogs [12]. This study also revealed that dog ownership was 22% more common in White European households than all other ethnicities included in the study [12]. The dog walkers among a population of 10–12-year-old children in Perth, Australia were more independently mobile and walked or played in the neighborhood, street, and yard at a significantly higher frequency when compared to the non-dog walkers, even though dog walk status was not found to be significantly associated with overall PA, walking, or pedometer steps [13]. Furthermore, subsequent analyses of the children 9–10 years old from the previously mentioned Liverpool SportsLinx Project failed to find evidence that children who live or walk with dogs are fitter or less likely to be obese than children who do not live or walk with dogs [14].

Dog ownership and walking has also been demonstrated to increase independent mobility and decrease anxiety levels in children. Given the research gaps that exist on youth dog ownership and youth dog walking as it correlates to youth PA, it is essential to understand the influence of built environment variables. One U.S.-based study, referenced previously, indicated that adolescents who lived in objectively walkable neighborhoods (e.g., lower perceived traffic safety, higher street connectivity, and less mixed use) were more likely to walk their dog [4]. This finding suggests that dog ownership and one’s perceived and objectively measured built environment may influence youth PA. With the majority of literature investigating the relationship between dog ownership and youth PA occurring outside of the U.S., there is a need to further analyze this association within the U.S., particularly among a sample of diverse preadolescent youth. Our study addressed this research gap and examined the influence of family dog ownership and parental built environment perceptions on PA behaviors among Washington, D.C. metropolitan (DMV) children.

## 2. Materials and Methods

### 2.1. Design and Sample

The Built Environment and Active Play (BEAP) Study questionnaire was mailed in September–December 2014 using a stratified sampling strategy to 2000 parents of children (7–12 years) living within nine DMV counties and cities (Washington, DC (District of Columbia); Fairfax County, Virginia (VA); Arlington County, VA; Loudon County, VA; Fairfax City, VA; Alexandria City, VA; Montgomery County, Maryland (MD); Prince George’s County, MD; and Frederick County, MD). The BEAP Study area map has been previously published [15].

All participants received the BEAP Study questionnaire, a $10 gift card, and a postage-paid self-addressed envelope with return instructions. If participants preferred to complete an online version of the questionnaire via Qualtrics.com, a secure and encrypted web address and unique access code were provided. Reminder and/or thank you post cards were mailed to the participants seven days after the initial mailing. Adapted from the Neighborhood Impact on Kids project survey, the BEAP Study questionnaire, a confidential study instrument, underwent several iterations of reliability and validity testing [15,16,17]. Within the questionnaire, several topic areas of questions such as child active play, active transportation, home and neighborhood built environment features, dog ownership, parental rules, demographics, and pre-existing health conditions were captured. An initial response rate of 10% was obtained, however, approximately 50 incomplete questionnaires were omitted from analysis. The final sample included 144 children. Implicit informed consent was obtained through the return of the completed questionnaire. The Institutional Review Board at The University of Maryland at College Park approved the study protocol (UMCP, 774586-1).

### 2.2. Built Environment Variables

Home was defined as the “home in which you and your child live” as well as the confirmed address to which the BEAP Study questionnaire was mail delivered. The questionnaire further defined home neighborhood as the area “within walking distance” or a “10–15 min walk from your home”. The three statement requests that were used to assess parental perceptions of the home neighborhood built environment and walkability were as follows: (1) “Please mark the answer that best applies to you and your child’s neighborhood”; (2) “My child can walk or bike to the closest local park or playground (alone or with someone) because:…”; and (3) “It is difficult for my child to be active in our home neighborhood because:…”. These three statement requests contained 44 subpart-responses in the form of statements or justifications (e.g., because there are sidewalks; because other children walk or bike) based on a four-point Likert scale of agreement, which were dichotomized and collapsed into “agree” and “disagree” responses. Additionally, parents were asked “Have you been the victim of a crime in your neighborhood?” and “Do you know someone who has been the victim of a crime in your neighborhood?”, which both elicited yes/no responses. A final question was included, “About how long would it take you to walk from your/your child’s home to each of the nearest places listed below?” This question contained 17 subpart-destinations (e.g., indoor recreation or exercise facility) with responses ranging from 1–5, 6–10, 11–20, 21–30, and over 30 min that were then dichotomized into “1–10 min” and “over 10 min”.

### 2.3. Dog Ownership Variables

Three questions were presented regarding family dog ownership, children walking the family dog, and children playing with the family dog. Parents were asked the binary question “Is there a dog in your home/child’s home?” If the parents responded yes to this question, they were then asked “How many days per week did your child spend walking your dog last week (including with a parent)?” and “How many days per week did your child spend playing outside with your dog last week (not including walking)?” These last two questions contained responses ranging from 1, 2, 3, 4, 5, 6, and 7 days.

### 2.4. Statistical Analysis

The Chi-square (**χ**^2^) Test of Independence was used to determine the independence or relationship of variables (H_o_ = There is no relationship between the two categorical variables). Hence, the differences in parental perceived built environment measures between children with and without family dogs were examined. Statistical analyses were carried out using STATA/MP 14.1 (College Station, TX, USA).

## 3. Results

The average age of the 144 children included in the BEAP Study population was 9.7 years (SD = 1.6). White children accounted for about half of the study population (56.3%), with African American (23.7%) and Asian American (10.4%) following as the highest represented groups. Based on parent-reported weights and heights, 25% of the children were classified as either overweight or obese. Approximately half (53%) of the participants were reported to live in households with an annual income greater than $100,000, but 14.7% of the subjects lived in households with an annual income less than $50,000.

**Figure 1:**
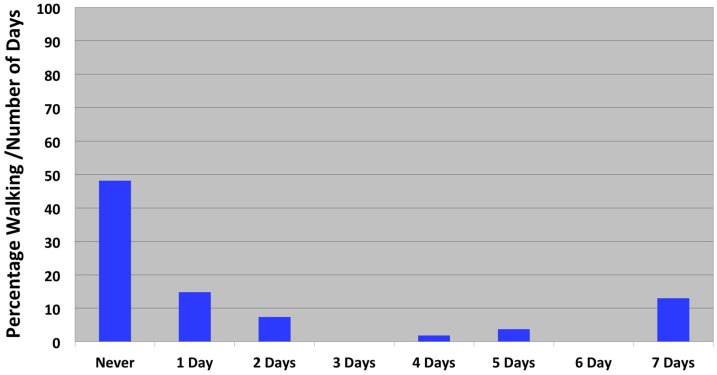
Frequency of children walking outdoors with family dog.

**Figure 2:**
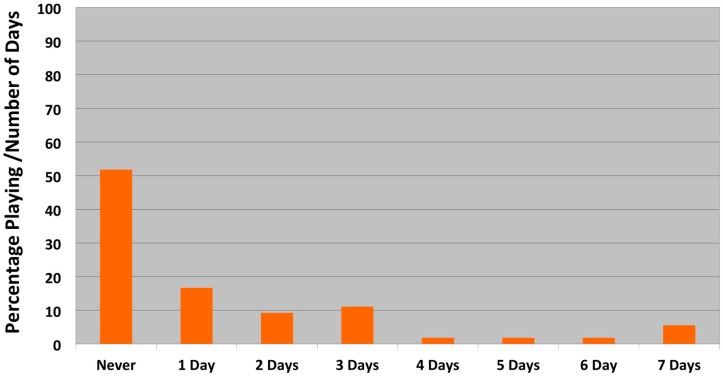
Frequency of children playing outdoors with family dog.

Among our study population, nearly 30% of the children lived in a household where there was also a family dog present, and of those children, all of them had parents with some college education or more (Table 1). The majority of dog owners were White (69.1%), compared to African American (14.3%), Other (9.5%), Asian American (7.1%), and Hispanic/Latino (4.7%) dog owners. Only 13% and 5.6% of the children walked and played outside with the dog daily, respectively (Figure 1 and Figure 2), and among these children, all of them were in first through fourth grades.

Chi-square (**χ**^2^) analysis was used to determine the differences in home built environment measures by dog ownership (Table 2). The majority of dog owners lived in single family homes (72.1%), compared to only 4.7% of dog owners residing in apartments. However, the majority of families that did not own a dog also lived in single family homes (61.4%). The distribution of front yard and back yard space is almost equal when comparing dog ownership. There were no statistically significant differences in home built environment features between families with dogs and families without dogs.

Differences in parental perceived built environment measures by dog ownership were also examined (Table 3). A significantly greater proportion (*p*-value < 0.05) of parents who owned dogs recognized and observed some home built environment measures that were PA-promoting for their children. Specifically, more parents who owned dogs agreed (strongly agree + agree) with built environment statements representing positive perceptions of some built environment features (e.g., neighborhood esthetics, safety, walkability infrastructure, and distance) as compared to the parents of children without dogs. For example, a greater proportion of dog owner parents (83.3%) agreed with the built environment measure representing their neighborhood walkability and safety, which stated that “The speed of traffic on most streets is usually 30 mph or less” compared to non-dog owner parents (67.7%). Additionally, no dog-owning parents thought that it was difficult for their child to be active in their home neighborhood because of crime, compared to parents who did not own dogs (10.0%). While statistical significance was not achieved, only 9.5% reported that it was likely their child “could be taken or hurt by a stranger” in their neighborhood as compared to the perceptions of 22.8% of non-dog owners.

When parents were asked to estimate the time it would take for them to walk from their home to the nearest destinations, there was no statistically significant difference of parental perception between dog owners and non-dog owners (Table 4). However, there were a few destinations that were found to be moderately different. It was found that there were less parents of children with dogs (9.5%) who perceived the distance to a fast food restaurant as a 10-min or less walk from their home, compared to the parents of children without dogs (26.0%). More dog owners (65.9%) perceived biking/hiking/walking trails and paths were a 10-min or less walk from their home, compared to non-dog owners (50.0%). Additionally, more dog owners (69.8%) compared to non-dog owners (57.3%) perceived public open space within a 10-min walk from their house.

## 4. Discussion

Our results suggest that dog ownership may provide more positive parental perceptions of the neighborhood built environment, which supports children’s outdoor PA through dog walking and playing. Unlike older adolescents or children outside of the U.S. who may experience more independent mobility, we found that parental perceptions of the built environment appear to influence their children’s dog-related PA.

Dog-owning parents had fewer perceived safety risks for their child being active in the neighborhood. This may be due to the fact that there is a higher intimacy level within the neighborhood because there is a higher degree of neighborhood engagement with dog walking and playing multiple times per day. Sense of place, including place attachment and place meaning, has been shown to shape the way individuals perceive their neighborhood and this can vary significantly among different individuals within the same neighborhood [18]. Activities, such as dog walking, foster neighborhood bond and intimacy, which ultimately increases place attachment and place meaning in a favorable manner. Parents may also believe that a dog can provide protection for their child and therefore they are less concerned with their child being hurt by a stranger. This may especially be true depending on the dog breed, as was found in the Liverpool SportsLinx Project. In this project, 9–10-year-old children who owned Pit Bulls as opposed to other non-Pit Bull breeds of dogs were more likely to report friends walking with their dog [11]. A general consensus has been reported that larger, louder, and darker dogs, such as Pit Bulls and Dobermans, would be helpful with house and neighborhood security because of their easily recognized physical appearance and reputation as attack- and guard-dogs [19].

Our results suggest that parents and children with dogs may visit different locations compared to those without dogs. For example, since public playgrounds are not appropriate places for dogs, dog owners may prefer open spaces where dogs can run off-leash. This was demonstrated in our research, where more dog owners perceived biking/hiking/walking trails and paths to be a 10-min or less walk from their home compared to non-dog owners.

There has been a challenge in establishing a causal influence of dog ownership on PA in adults, and this certainly has not been established in adolescents or children. While longitudinal studies in adults have suggested that dog owners become more active, the effect in children remains unknown partly because this research has not been fully explored. Consistent with U.S. households, 30% of our study population owned dogs and among this sample, all of the parents with the exception of one received some college education or more. Therefore, this more educated population may have underestimated the potential influence of dog ownership on PA since this sample of parents may appreciate the importance of PA for their children, regardless of dog ownership. Furthermore, nearly 70% of our study population who owned dogs was White, which was similar to previous findings in other research [4,5,12]. While non-Whites are much less likely to own pets compared to Whites, the human–dog companionship has also been shown to vary by race and ethnicity [20,21]. Among the exceptionally limited research exploring the relationship between ethnic diversity and companion animals, it has been found that various groups of non-Whites were afraid of dogs, disliked the hygienic mannerisms of dogs, or used their dog for personal safety [20,21]. Therefore, the motivation for dog ownership may not be founded in reasons of companionship, which may limit the PA interactions of walking or playing for adults and children.

As a strength of this study, to date, this is the first U.S.-based study to explore the relationship of dog ownership and PA among a racially and ethnically diverse population of younger children. A prior U.S.-based study in younger children that did report increased PA with dog ownership consisted of over 97% White children [6]. However, the current study consisted of less than 60% White children. In our research, it was found that parental perceptions of the built environment could have a meaningful impact on children's dog-related PA. However, additional research is needed to understand the social and cultural influences of this impact.

As with many studies, this study encountered a few limitations. Specifically, the questionnaire relied on family dog ownership as the primary measure. It is possible that children could walk or play regularly with other dogs in the neighborhood, but this would not be accounted for since the family would not own that dog. The age and type of dog could also impact the daily walking and playing habits for children, as these factors influence the dog’s energy levels and frequency of needing outside relief. Furthermore, only outdoor playing was assessed and data on indoor dog playing was not captured in this study. Another important limitation of this study is that the questionnaire only collected data on how many days per week the child walked or played with the dog and it did not ask about the minutes or intensity of the PA. Therefore, dog ownership contribution to daily PA accumulation could not be objectively or subjectively estimated. Additionally, since cross-sectional studies only provide a snap-shot in time, youth PA patterns prior to and during dog ownership could not be established. Again, additional research in this area of study, which would collect both objective and subjective PA, is warranted since dog walking or playing could provide an integral outlet for increasing youth PA.

## 5. Conclusions

This study suggests that dog ownership may positively influence parental perceptions of the neighborhood built environment. This is important because this positive perception may facilitate their children’s outdoor PA through dog walking and playing.

## Figures and Tables

**Table 1:** Built environment and active play (BEAP) study child participant demographics.

Parameter	Total *n* (%)	No Own Dog *n* (%)	Yes Own Dog *n* (%)	Daily Outdoor Dog Walking— *n* (%)	Daily Outdoor Dog Playing— *n* (%)
Gender					
Male	72 (50.0)	48 (47.5)	24 (55.8)	2 (28.6)	0
Female	72 (50.0)	53 (52.8)	19 (44.2)	5 (71.43)	3 (100.0)
Ethnicity/Race					
Hispanic/Latino	7 (4.9)	5 (5.1)	2 (4.7)	0	0
African American	32 (23.7)	26 (28.0)	6 (14.3)	3 (42.9)	1 (33.3)
American Indian/Alaska Native	1 (0.7)	1 (1.1)	0	0	0
Asian American	14 (10.4)	11 (11.8)	3 (7.1)	0	0
White	76 (56.3)	47 (50.5)	29 (69.1)	4 (57.1)	2 (66.7)
Other	12 (8.9)	8 (8.6)	4 (9.5)	0	0
Highest Grade Completed					
1st Grade	14 (9.8)	10 (10.0)	4 (9.3)	2 (28.6)	1 (33.3)
2nd Grade	24 (16.8)	17 (17.0)	7 (16.3)	2 (28.6)	0
3rd Grade	21 (14.7)	17 (17.0)	4 (9.3)	1 (14.3)	0
4th Grade	24 (16.8)	12 (12.0)	12 (27.9)	2 (28.6)	2 (66.7)
5th Grade	34 (23.8)	28 (28.0)	6 (14.0)	0	0
6th Grade	17 (11.9)	9 (9.0)	8 (18.6)	0	0
>6th Grade	9 (6.3)	7 (7.0)	2 (4.7)	0	0
Annual Household Income					
≤$30,000	6 (4.4)	4 (4.2)	2 (4.9)	1 (16.7)	1 (33.3)
$30,001–$50,000	14 (10.3)	13 (13.7)	1 (2.4)	0	0
$50,001–$75,000	12 (8.8)	7 (7.4)	5 (12.2)	0	0
$75,001–$100,000	20 (14.7)	13 (13.7)	7 (17.1)	2 (33.3)	1 (33.3)
$100,001–$150,000	27 (19.9)	21 (22.1)	6 (14.6)	0	0
$150,001–$250,000	29 (21.3)	18 (19.0)	11 (26.8)	2 (33.3)	1 (33.3)
$250,001–$500,000	13 (9.6)	9 (9.5)	4 (9.8)	1 (16.7)	0
>$500,000	3 (2.2)	2 (2.1)	1 (2.4)	0	0
Parent Education					
Some High School	1 (0.7)	1 (1.0)	0	0	0
Completed High School	7 (4.9)	6 (6.1)	1 (2.3)	0	0
Some College	31 (21.8)	22 (22.2)	9 (20.9)	2 (28.6)	2 (66.7)
Completed College	47 (33.1)	32 (32.3)	15 (34.9)	2 (28.6)	0
Completed Graduate School	56 (39.4)	38 (38.4)	18 (41.9)	3 (42.9)	1 (33.3)
Doctor Diagnosed Illness					
Anxiety	9 (6.5)	4 (4.1)	5 (12.2)	0	0
Asthma	25 (17.6)	19 (18.8)	6 (14.6)	2 (33.3)	0
ADHD/ADD	17 (12.0)	10 (10.0)	7 (16.7)	1 (14.3)	0
Depression	2 (1.4)	2 (2.0)	0	0	0
High Blood Pressure	1 (0.7)	0	1 (2.4)	0	0
High Cholesterol	3 (2.1)	2 (2.0)	1 (2.4)	1 (16.7)	1 (33.3)
Overweight/Obese	11 (7.9)	9 (9.1)	2 (5.0)	0	0
Child Weight Status *					
Underweight	12 (13.3)	7 (10.6)	5 (20.8)	0	0
Healthy Weight	55 (61.1)	43 (65.2)	12 (50.0)	2 (66.7)	1 (50.0)
Overweight	12 (13.3)	9 (13.6)	3 (12.5)	1 (33.3)	0
Obese	11 (12.2)	7 (10.6)	4 (16.7)	0	1 (50.0)
Born in United States					
Yes	134 (95.0)	93 (93.9)	41 (97.6)	6 (100.0)	2 (66.7)
No	7 (5.0)	6 (6.1)	1 (2.4)	0	1 (33.3)
County Residence					
Montgomery County	38 (27.1)	27 (27.6)	11 (26.2)	3 (42.9)	1 (33.3)
Fairfax County	39 (27.9)	29 (29.6)	10 (23.8)	1 (14.3)	0
Loudoun County	19 (13.6)	10 (10.2)	9 (21.4)	1 (14.3)	0
Prince George’s County	20 (14.3)	16 (16.3)	4 (9.5)	1 (14.3)	0
Frederick County	10 (7.1)	7 (7.1)	3 (7.1)	0	1 (33.3)
Washington, DC	14 (10.0)	9 (9.2)	5 (11.9)	1 (14.3)	1 (33.3)

* Child Weight Status calculated based on parent-reported child weight and height, and weight classifications were based on Centers for Disease Control and Prevention BMI-for-age weight status categories.

**Table 2:** Differences in home built environment measures by family dog ownership.

Destinations within 1–10 Min Walk of Home	Yes Dog (%)	No Dog (%)	χ^2^	*p*-Value ^○^
Home Type			3.0	0.400 ^∆^
Single Family Home	72.1	61.4		
Townhouse	23.3	24.8		
Apartment	4.7	12.9		
Other	0	1.0		
Front Yard	83.7	82.2	0.050	0.823
Back Yard	83.7	80.0	0.272	0.602
Side Yard	60.5	52.5	0.763	0.382
Driveway	72.1	67.0	0.362	0.548
Home Street Sidewalks	72.4	76.2	0.054	0.816
Neighborhood Sidewalks	76.7	83.2	0.817	0.366
Home Ownership			2.5	0.293
Own	72.1	72.3		
Rent	20.9	25.7		
Home Street Dead End/Cul-de-sac	30.2	34.6	0.265	0.607 ^○^

^○^ 1 Degree of freedom unless noted otherwise; ^∆^ 3 Degrees of freedom.

**Table 3:** Differences in perceived built environment measures by family dog ownership.

Built Environment Measure	Yes Dog Agree (%)	No Dog Agree (%)	χ^2^	*p*-Value ^○^
Many streets in my neighborhood are hilly.	51.6	51.5	0.002	0.969
There are not any dead end streets.	21.4	24.0	0.110	0.741
Sidewalks are on most streets.	76.2	82.2	0.675	0.411
Usually sidewalks are separated from the road/traffic by parked cars.	43.9	61.6	3.7	0.054 ^❖^
Trees are along the streets.	88.4	89.0	0.119	0.913
My child can look at many interesting things while walking.	81.4	77.0	0.342	0.559
There are many natural things for my child to see.	81.4	86.0	0.490	0.484
Many buildings/homes are present for my child to see.	83.7	82.8	0.017	0.896
The traffic makes it difficult or unsafe for my child to walk.	27.9	41.8	2.5	0.116
The speed of traffic on most streets is usually 30 mph or less.	83.3	67.7	3.6	0.057 ^❖^
Most motorist drive faster than the posted speed limits.	79.1	82.0	0.169	0.681
Streets have good lighting at night.	51.2	55.5	0.223	0.637
Walkers and bikers can be easily seen by people in their homes.	72.1	72.0	<0.001	0.991
Busy streets have crosswalks and signals.	72.1	71.0	0.018	0.895
There is a high crime rate.	11.6	18.8	1.1	0.290
The streets have a lot of litter.	14.0	13.9	<0.001	0.988
Many families look like us in our neighborhood.	66.7	71.9	0.380	0.538
You have been the victim of a crime in your neighborhood.	14.0	22.0	1.2	0.266
You know someone who has been the victim of a crime in your neighborhood.	44.2	49.5	2.0	0.363
I’m afraid of my child being taken or hurt by…				
a stranger when he/she is outside without me.	51.2	54.5	0.131	0.717
a known “bad” person (adult or child) in my neighborhood.	14.0	18.0	0.353	0.553
It is likely that my child can be taken or hurt by a stranger…				
in my neighborhood.	9.5	22.8	3.4	0.065
in my yard, driveway, or common area.	7.0	12.9	1.1	0.303
My child can walk or bike to the closest park or playground because…				
there are sidewalks or bike lanes.	74.4	76.0	0.041	0.840
the route is simple.	81.4	82.0	0.007	0.931
the route has good lighting when it’s dark outside.	44.2	37.8	0.516	0.472
there are no dangerous crossings.	48.8	48.5	0.002	0.969
my child does not get too hot and sweaty.	51.2	54.6	0.145	0.704
other children walk or bike.	81.4	75.8	0.546	0.460
it is considered cool to walk or bike.	61.9	63.9	0.051	0.821
my child does not have much stuff to carry.	72.1	79.6	0.958	0.328
it is easier than me driving there on the way to something else.	52.4	40.2	1.8	0.184
it involves very little planning ahead.	69.8	67.4	0.081	0.777
there are areas to leave a bike safely.	60.5	57.6	0.103	0.748
there are no stray dogs.	76.7	62.6	2.7	0.100
it is not too far.	83.3	83.7	0.003	0.960
my child would not have to walk/bike through high crime or unsafe areas.	81.4	73.7	0.965	0.326
It is difficult for my child to be active in our home neighborhood because…				
there is no choice of activities.	19.1	17.2	0.071	0.790
there is no play equipment (e.g., basketball hoop).	19.1	19.0	<0.001	0.995
there is no adult supervision.	24.4	24.7	0.002	0.965
there are no other children there.	28.6	23.0	0.494	0.482
it is not safe because of crime.	0	10.0	4.52	0.034 ^❖^
it is not safe because of traffic.	11.9	25.3	3.14	0.077
it does not have good lighting.	26.2	30.3	0.242	0.623

^○^ 1 Degree of freedom unless noted otherwise; ^❖^ Statistically significant (*p*-value < 0.05).

**Table 4:** Differences in parental perceived walking time by family dog ownership.

Destinations within 1–10 Min Walk of Home	Yes Dog (%)	No Dog (%)	χ^2^	*p*-Value ^□^
Friend’s or relative’s house	72.5	70.3	0.074	0.963
Public playground	64.3	71.0	0.878	0.645
Biking/hiking/walking trails and paths	65.9	50.0	3.35	0.187
Basketball court	62.8	64.0	1.2	0.546
Public open space that is not a park	69.8	57.3	3.5	0.175
Public park	53.5	57.1	0.164	0.921
Bus or Metro stop	47.6	54.0	0.781	0.677
Outdoor swimming pool	48.8	33.0	4.0	0.133
Other playing fields/court (e.g., tennis, softball)	37.2	44.4	1.00	0.606
School grounds during non-school hours	48.8	42.4	0.873	0.646
Convenience/corner store	20.9	34.0	2.7	0.260
Fast food restaurant	9.5	26.0	4.9	0.088
Indoor recreation or exercise facility (e.g., YMCA)	11.6	10.2	0.535	0.765
Beach, lake, river or creek	14.0	11.1	0.230	0.891
Library	14.3	8.1	2.5	0.291
Ski or other winter recreation area	0	1.0	0.440	0.802
Indoor swimming pool	12.2	6.0	1.6	0.457

**^□^** 2 Degree of freedom unless noted otherwise.
